# Sex-Specific Timelines for Adaptations of Prefrontal Parvalbumin Neurons in Response to Stress and Changes in Anxiety- and Depressive-Like Behaviors

**DOI:** 10.1523/ENEURO.0300-22.2023

**Published:** 2023-03-01

**Authors:** Emma Woodward, Claudia Rangel-Barajas, Amanda Ringland, Marian L. Logrip, Laurence Coutellier

**Affiliations:** 1 Department of Neuroscience, The Ohio State University, Columbus, Ohio 43210; 2 Department of Psychology, The Ohio State University, Columbus, Ohio 43210; 3 Department of Psychology, Indiana University–Purdue University Indianapolis, Indianapolis, Indiana 46202; 4 Stark Neurosciences Research Institute, Indiana University School of Medicine, Indianapolis, Indiana 46202

**Keywords:** chronic stress, parvalbumin neurons, prefrontal cortex, sex differences, vulnerability

## Abstract

Women are twice as likely as men to experience emotional dysregulation after stress, resulting in substantially higher psychopathology for equivalent lifetime stress exposure, yet the mechanisms underlying this vulnerability remain unknown. Studies suggest changes in medial prefrontal cortex (mPFC) activity as a potential contributor. Whether maladaptive changes in inhibitory interneurons participate in this process, and whether adaptations in response to stress differ between men and women, producing sex-specific changes in emotional behaviors and mPFC activity, remained undetermined. This study examined whether unpredictable chronic mild stress (UCMS) in mice differentially alters behavior and mPFC parvalbumin (PV) interneuron activity by sex, and whether the activity of these neurons drives sex-specific behavioral changes. Four weeks of UCMS increased anxiety-like and depressive-like behaviors associated with FosB activation in mPFC PV neurons, particularly in females. After 8 weeks of UCMS, both sexes displayed these behavioral and neural changes. Chemogenetic activation of PV neurons in UCMS-exposed and nonstressed males induced significant changes in anxiety-like behaviors. Importantly, patch-clamp electrophysiology demonstrated altered excitability and basic neural properties on the same timeline as the emergence of behavioral effects: changes in females after 4 weeks and in males after 8 weeks of UCMS. These findings show, for the first time, that sex-specific changes in the excitability of prefrontal PV neurons parallel the emergence of anxiety-like behavior, revealing a potential novel mechanism underlying the enhanced vulnerability of females to stress-induced psychopathology and supporting further investigation of this neuronal population to identify new therapeutic targets for stress disorders.

## Significance Statement

While adult women are more often diagnosed with a mood disorder after facing stressful events than men, the mechanisms responsible for this sex-specific vulnerability remain unknown. This study uses a mouse model of stress-induced anxiety-like and depressive-like behaviors to show a novel mechanism, adaptation of prefrontal parvalbumin (PV) neurons, that may underlie the greater susceptibility of females to stress-related psychopathologies. Female parvalbumin neurons adapt after shorter periods of chronic stress than male neurons, paralleling earlier emergence of anxiety-like behaviors in females. We propose that prefrontal PV interneurons are an important contributor to female stress sensitivity that should be further investigated to identify new therapies for psychopathology related to stress.

## Introduction

Exposure to stress is a triggering factor for emotional dysregulation, including anxiety and depression (Pittenger and Duman, 2008). The extent to which stress leads to changes in emotional behaviors varies between individuals along a spectrum from full resilience to high vulnerability. One of the major susceptibility factors to stress-induced emotional dysregulation is sex, with depression and anxiety affecting women at twice the rate of men (Kessler et al., 2005). Not only are women at increased risk for developing these conditions (Kendler, 1998; Kessler, 2003; Armstrong et al., 2018), but they often display more severe symptoms (de Graaf et al., 2002; Schoevers et al., 2003). Studies to identify factors that drive vulnerability to stress-induced psychopathology have largely focused on male subjects, leading to an overall lack of knowledge about the molecular mechanisms underlying individualized, sex-specific maladaptive responses to stress. Elucidating these mechanisms must remain a priority to improve the diagnosis and prevention of stress-related mental illnesses, particularly toward developing personalized treatments for these disorders.

Recent work has begun to elucidate sex differences in neuroadaptations to stress that may underlie differential disease prevalence. Sex-specific gene expression patterns have been identified in regions of the brain involved in stress responses, including the nucleus accumbens and prefrontal cortex (Hodes et al., 2015; Barko et al., 2019). For example, chronic stress increased GABA-related and glutamate-related gene expression in the medial prefrontal cortex (mPFC) of female, but not male, mice (Barko et al., 2019). These findings parallel human studies of major depressive disorder showing sexual dimorphism at the transcriptional level in brain regions regulating emotions (Labonté et al., 2017; Scarpa et al., 2020) and emphasize that the male and female brains respond differently to stress, leading to sex-specific vulnerabilities to stress-related psychopathologies.

Changes within the mPFC have been associated with stress vulnerability. Increased mPFC activity during stress has been associated with stress resilience in both humans and rodent models (Amat et al., 2006, 2008; Sinha et al., 2016), whereas decreased neuronal activity during stress was associated with a vulnerable phenotype (Vialou et al., 2014). Rodent studies have revealed sexually divergent adaptation of male and female mPFC after stress (Moench and Wellman, 2017), as well as a specific role for estrogen in those adaptations (Garrett and Wellman, 2009; Shansky et al., 2010), suggesting that the mPFC could be a key site to study sex-specific vulnerability to stress. Importantly, studies have shown that female mice with reduced prefrontal expression of the neuronal activity marker c-Fos after unpredictable chronic mild stress (UCMS) also displayed more depressive-like and anxiety-like behaviors (Shepard et al., 2016; Shepard and Coutellier, 2018). While those studies did not distinguish between different neuronal subtypes, work focusing on parvalbumin-expressing (PV^+^) interneurons indicated that those neurons express more c-Fos after UCMS (Page et al., 2019). This would suggest that greater activation of PV^+^ neurons in the PFC increases the inhibition of excitatory cells, leading to an overall decrease in c-Fos expression. Indeed, the mPFC contains a heterogeneous population of excitatory pyramidal principal neurons and inhibitory GABAergic interneurons, which regulate circuit activity. Among prefrontal interneurons, those expressing PV are key regulators of the firing pattern of principal projection neurons and are highly sensitive to stress (Maguire, 2014; Page et al., 2019). Altered excitability of PV^+^ interneurons following chronic stress would thus change the activity of prefrontal neurons projecting to subcortical and limbic regions, thereby contributing to aberrant emotional regulation. In support of this idea, PV^+^ neuron number was reduced, and the dendritic length of pyramidal neurons increased in layers II/III of mPFC after antidepressant treatment in male rats (Song et al., 2019). Conversely, the increased number of mPFC PV^+^ neurons expressing c-Fos after UCMS was positively correlated with heightened anxiety-like behaviors in female mice (Shepard et al., 2016; Page et al., 2019). Together, these studies suggest a role for mPFC PV^+^ neurons in gating anxiety-like behaviors, and sex-specific alterations in these neurons in response to chronic stress could regulate differential vulnerability to stress-induced anxiety and depression.

We hypothesized that UCMS would change mPFC PV^+^ neurons in a sex-specific manner, with altered excitability of prefrontal cortical PV^+^ neurons and anxiety-like behavior emerging after fewer UCMS exposures in females than in males. Additionally, we hypothesized that activating mPFC PV^+^ neurons throughout the UCMS exposure would shift the natural resilience of males to UCMS, causing some anxiety-like behaviors to emerge after the shorter UCMS duration. To test these hypotheses, mice were exposed to UCMS for 4 or 8 weeks, without or with chemogenetic activation of mPFC PV^+^ neurons, and alterations in anxiety-like and depressive-like behaviors were assessed. Additionally, whole-cell patch-clamp recordings from infralimbic (IL) layer II/III PV^+^ neurons from both sexes measured the effects of UCMS on neuronal excitability and basic neuronal properties.

## Materials and Methods

### Animals

Adult male and female mice were group housed by sex (two to five mice per cage, unless specified otherwise), provided *ad libitum* access to food and water, and maintained on a 12 h reverse light/dark cycle. C57BL/6J mice (The Jackson Laboratory) were used for chronic stress exposure and immunofluorescent staining experiments. PV:TdTomato mice generated by mating PV:Cre and Ai14 [B6.Cg-Gt(ROSA)26Sortm14(CAG-tdTomato)Hze/J] mice (founders from The Jackson Laboratory; Jones and Sheets, 2020) were used for electrophysiological studies. PV:Cre mice [B6;129P2-Pvalbtm1(cre)Arbr/J; The Jackson Laboratory] were used for chemogenetic experiments. All experiments were conducted in accordance with protocols approved by the Institutional Animal Care and Use Committees of The Ohio State University and Indiana University–Purdue University Indianapolis, and with the National Institutes of Health *Guide for the Care and Use of Laboratory Animals*.

### Unpredictable chronic mild stress procedure

Adult mice (at least 9 weeks old) were single housed and exposed to a daily mild stressor according to an unpredictable schedule for 4 or 8 weeks. Stressors included a 24 h absence of nesting material, an 8 h absence of bedding, a 6 h cage tilt, an 8 min restraint stress in the dark, and a 4 min restraint stress under bright light. The timeline of stressors throughout the UCMS protocol is provided in Table 1. The protocol was modified from that developed for male mice by Monteiro et al. (2015) to include stressors that are applicable to both males and females. Specifically, we removed the social defeat stressor and included other stressors that have been previously shown to induce changes in anxiety-like and depressive-like behaviors, in a sex-dependent and behavioral assay-dependent manner (Mineur et al., 2006; Shepard et al., 2016; Page et al., 2019). Control animals were group housed and were handled once daily for 1–2 min throughout the UCMS period.

**Table 1:** Timeline of stressors followed for the 4 week UCMS protocol

Day	Stressor
1	CT
2	BR
3	EC
4	CT
5	RN
6	DR
7	CT
8	RN
9	DR
10	RN
11	BR
12	CT
13	BR
14	EC
15	DR
16	CT
17	RN
18	DR
19	RN
20	BR
21	EC
22	CT
23	EC
24	BR
25	RN
26	BR
27	RN
28	DR

The same timeline was repeated for the 8 week UCMS protocol. CT, 20° cage tilt along the vertical axis for 6 h; BR, tube restraint under bright light conditions for 4 min; EC, empty cage (absence of nesting) for 8 h; RN, remove nesting for 24 h; DR, tube restraint in dark conditions for 8 min.

### Behavioral testing

To assess anxiety-like behaviors, elevated plus maze (EPM), open field (OF), and marble-burying tests were used. To measure depressive-like behaviors, we used the splash test, and to measure changes in response to stimuli with negative valence, we used an adapted version of conditioned placed aversion (CPA) to a low dose of lithium chloride (LiCl). All behavioral assays were conducted during the dark phase and started 24 h after the last UCMS/handling session. On testing days, mice were allowed a 1 h habituation period in the testing room before the beginning of testing. Only one test was conducted per day, with the least aversive test conducted first (CPA test was the last test conducted because of repeated intraperitoneal injections). All tests were recorded with an overhead camera for offline analysis. Automated, unbiased analyses were conducted using EthoVision XT software (Noldus Information Technology) for EPM, OF, and CPA tests. Grooming and burying behaviors in the splash and marble-burying tests, respectively, were scored by hand by a trained experimenter blinded to the experimental groups.

#### Elevated plus maze

The EPM apparatus consisted of a raised (74 cm) plus-shaped arena with four arms (length of each, 34 cm). The two closed arms were surrounded by 22-cm-high black walls. Mice were allowed to explore the maze for 5 min. Testing occurred under red light. At the end of the test, mice were returned to their home cage and the arena was cleaned with 70% ethanol before testing the next mouse.

#### Open field test

The OF apparatus consisted of a 40 × 40 cm square gray arena with opaque white walls. Mice were allowed to explore the arena for 10 min under dim white light (50 lux). They were then returned to their home cage and the arena was cleaned with 70% ethanol.

#### Lithium chloride-conditioned place aversion

The CPA test was adapted from Ventura et al. (2013). The test takes place in a two-chamber apparatus with a central alley, with distinct patterns on the walls of each chamber. CPA training occurred over 10 d. On day 1 (pretest), mice were placed individually in the apparatus with all doors open and were allowed to explore freely for 20 min. Next, mice were conditioned to associate one chamber with LiCl injection (63.5 mg/kg, i.p.), and the other chamber with saline injection. This low dose of LiCl induces aversion in stressed mice but not in control mice (Ventura et al., 2013), therefore allowing us to determine whether UCMS-exposed mice have increased sensitivity to aversive stimuli. Conditioning sessions were each 40 min long, with LiCl and saline conditioning on alternate days (four pairings per chamber/treatment). On day 10, testing for the expression of CPA was conducted, with mice placed individually in the apparatus with all doors open and allowed to explore freely for 20 min. An aversion score is calculated as the difference in time spent in the LiCl-associated chamber between the test session (day 10) and the pretest session (day 1). A negative score indicates aversion for the LiCl-associated chamber.

#### Marble-burying test

Mice were placed in a new, clean cage with 10 cm of clean corncob bedding, with 20 marbles evenly distributed on top of the bedding throughout the cage area. Mice were allowed to freely explore the cage for a 30 min period, at the end of which the number of marbles buried was recorded (any marble more than half covered with bedding was considered as buried). The test took place under dim white light (50 lux). Each mouse was then returned to its home cage.

#### Splash test

Mice were placed in a new, clean cage with fresh bedding and allowed to habituate for 10 min. Following habituation, a 10% sucrose solution was sprayed on the back of the animal. Grooming behavior was recorded directly over a 5 min period. Mice were then returned to their home cage.

### Immunofluorescent staining

A new cohort of mice was used to determine the effect of UCMS exposure on the activity of PV cells. Twenty-four hours after the last stressor or handling session, brains were collected via perfusion with 4% cold paraformaldehyde (PFA; *N* = 4–5/group). Brains were removed and kept in 4% PFA at 4°C overnight before storage in a sucrose solution (30% sucrose). Brains were frozen on dry ice and sectioned at 50 μm using a cryostat. Immunohistochemistry was performed to identify prefrontal PV^+^ neurons expressing the marker of chronic activity, ΔFosB, the stable splice variant of FosB, which accumulates with long-term stimulation because of its long half-life. Free-floating staining was performed on every third section using a guinea pig anti-PV antibody (1:500; product #195004, Synaptic Systems) and a rabbit recombinant anti-FosB/ΔFosB antibody (1:2000; catalog #Ab184938, Abcam), followed by incubation with fluorescent secondary antibodies. Quantitative analysis of PV^+^ cells expressing FosB/ΔFosB in the mPFC was achieved using the unbiased stereology method with StereoInvestigator software (MBF Bioscience). Cells were counted in every third section by an experimenter blind to the experimental groups. A total of three sections per animal were analyzed, and regions of interest [prelimbic (PrL) PFC, IL PFC] were delineated according to the *Mouse Brain Atlas* by Franklin and Paxinos (2008). Accuracy of the estimate of the total number of positive-stained cells based on our counting was assured by verifying that the mean coefficient of error of Gundersen was <0.10. The primary antibody used to stain for ΔFosB does not discriminate between FosB and ΔFosB, but chronic exposure to stimuli desensitizes acute FosB inducibility. Thus, the staining reflects differences in ΔFosB accumulation (García-Pérez et al., 2012). The percentage of PV cells expressing FosB was computed for each animal and used for statistical analyses.

### Chemogenetic manipulation of prefrontal PV^+^ cells

Adeno-associated virus (AAV) designer receptors exclusively activated by designer drug (DREADD) vectors (Addgene) were stereotaxically infused in the ventromedial PFC (including the central part of the PrL and IL cortices) of male PV:Cre mice. Briefly, AAV2/hSyn-DIO-hm3D(Gq)-mCherry (“hM3DGq”) was injected bilaterally (0.5 μl/side, ∼10^12^ viral genomes/ml) into mPFC using the following coordinates: anteroposterior, +1.7 mm; mediolateral, ±0.2 mm; dorsoventral, −2.6 mm. AAV2/hSyn-DIO-mCherry was used as the control virus. Previous work demonstrated that chemogenetic activation of PV^+^ neurons from both PrL and IL regions increases anxiety-like behaviors in female mice (Page et al., 2019). To be able to compare our findings with this study, we opted to use a similar strategy. Mice remained undisturbed for at least 21 d after surgery to allow full expression of the DREADD virus in PV^+^ cells before the commencement of behavioral testing. All mice received a daily intraperitoneal injection of clozapine-*N*-oxide (CNO; 0.5 mg/kg) 30 min before daily handling or stressor throughout the UCMS period. After the completion of behavioral assays, viral injection sites were verified using a rabbit anti-DsRed antibody (1:1000; TaKaRa Bio) followed by an Alexa Fluor anti-rabbit 555 secondary antibody (1:500; Thermo Fisher Scientific) to target mCherry in prefrontal brain sections.

### Whole-cell patch-clamp electrophysiology

#### Brain slice preparation

Mice were decapitated under deep isoflurane anesthesia, and the brain was immediately removed and placed in oxygenated (95% O_2_/5% CO_2_), ice-cold cutting solution consisting of the following (in mm): 194 sucrose, 30 NaCl, 4.5 KCl, 1.2 NaH_2_PO_4_, 26 NaHCO_3_, 1 MgCl_2_, and 10 glucose, at pH 7.4 and ∼310 mOsm. Brain slices containing mPFC were cut using a vibrating-blade microtome (model VT 1200S, Leica Biosystems). Immediately after dissection, slices were kept at room temperature for a resting period of at least 1 h in artificial CSF (aCSF) solution containing the following (in mm): 127 NaCl, 2.5 KCl, 25 NaHCO_3_, 1.25 NaH_2_PO_4_, 2 CaCl_2_, 1 MgCl_2_, and 25 glucose, at pH 7.4 and ∼290 mOsm.

#### Electrophysiological recordings

After the resting period, individual slices were transferred to a submerged recording chamber fixed to the stage of an upright microscope and continuously perfused with oxygenated aCSF (2 ml/min). Patch micropipettes (resistance, 3–6 MΩ) were fabricated from borosilicate glass capillaries (outer diameter, 1.5 mm; inner diameter, 1.12 mm) using a horizontal puller (model P-1000, Sutter Instruments). Recording pipettes were filled with a potassium gluconate (KG)-based internal solution consisting of the following (in mm): 126 KG, 4 KCl, 10 HEPES, 4 MgATP, 0.3 NaGTP, and 10 phosphocreatine, at pH 7.4 and 280 mOsm. Whole-cell recordings were obtained from PV^+^ neurons in layers II/III of the IL subdivision of mPFC using current-clamp mode. Initially, to confirm viability, neurons were visualized using infrared differential interference contrast (IR-DIC) optics via a CCD camera (ORCA-Flash CMOS, Hamamatsu) with a 40× water-immersion objective; to identify PV^+^ neurons by TdTomato fluorescence, neurons were illuminated by solid-state white light excitation (Sola Light Engine). Recordings were obtained using a MultiClamp 700B amplifier and a Digidata 1550B digitizer (Molecular Devices). All recordings were filtered at 2 kHz and digitized at 10 kHz. Data were acquired using Clampex 11 software (Molecular Devices). After patching, neurons were allowed to equilibrate to the whole-cell configuration for at least 5 min before collecting data. Access resistance ranged from 10 to 25 MΩ and was monitored throughout the experiment; recordings with changes in access resistance >20% were not included in the data analysis. Following equilibration, neuronal excitability was recorded by applying step current injections (range, −200 to 400 pA; 50 pA increments) for 500 ms intervals.

#### Data analysis

Action potential (AP) detection and measurements of AP parameters were performed using the threshold search module implemented in Clampfit 11 (Molecular Devices). The excitability parameters recorded included resting membrane potential (RMP), AP threshold, AP peak amplitude, AP half-width, input resistance, and frequency. AP threshold, peak amplitude, and peak half-width were analyzed with data collected from the first current step that produced APs. AP height was calculated as previously described (Bean, 2007; Brickley et al., 2007). Briefly, the spike height of a sequence of the three initial APs was calculated at both threshold and suprathreshold depolarizing current steps. The AP height was defined as the peak relative to the most negative voltage reached during the afterhyperpolarization immediately after the spike for analysis. AP height was normalized to the first AP and averaged across neurons from the control group (number of neurons N = 14 male, 19 female) and compared with the AP height of the UCMS groups (4 weeks of UCMS neuron ns = 11 males, 17 females; 8 weeks of UCMS neuron ns = 17 male, 15 female) for visual depiction.

### Statistical analyses

Data were analyzed using Prism (GraphPad Software) or SPSS (IBM). Behavioral (Table 2) and immunohistochemical (Table 3) data were analyzed using two-way ANOVAs, with sex (male vs female) and UCMS (0, 4, or 8 weeks) as the factors. Chemogenetic data were also analyzed by two-way ANOVAs, with UCMS (0 vs 4 weeks) and DREADD (Gq vs control) as the factors (Table 4). Electrophysiological analysis of AP firing (Table 5), measured across multiple stimulus intensities, was performed using a three-way ANOVA with repeated measures, with the between-subjects factors of sex (male vs female) and UCMS (0, 4, or 8 weeks), and the within-subjects factor stimulus intensity. Because neurons did not fire APs at negative current intensities (i.e., hyperpolarized states) and <6% of neurons fired APs spontaneously at baseline (0 pA injection; 5 of 93 cells recorded), we excluded these minimally variant data from ANOVAs, only including intensities >0 pA. Despite excluding nonvariant and minimally variant stimulus intensities, data analyzed by three-way ANOVA were heteroskedastic, violating one of the assumptions of ANOVA. To eliminate this repeated variable, AP firing data across all current intensities were transformed to the area under the curve and analyzed by two-way ANOVA, with the between-subjects factors UCMS and sex. Additionally, because of the complexity of interpreting interactions in three-way ANOVAs, planned within-sex analyses by two-way ANOVAs with repeated measures (between-subjects factor, UCMS; within-subjects factor, stimulus intensity) were conducted for excitability data. Changes in AP height also were analyzed using three-way ANOVAs, with the within-subjects factor AP number and the between-subjects factors sex (male vs female) and UCMS (0, 4, or 8 weeks). Although data are visually depicted normalized to the first spike at threshold, statistical analyses were performed on raw data so that equal variation existed among the three APs for the threshold group. As for AP firing, these analyses were followed up by planned within-sex analyses (Table 5). Neuronal parameters (Table 6) were analyzed using two-way ANOVAs with sex (male vs female) and UCMS (0, 4, or 8 weeks) as the factors. When necessary because of sphericity violations, the Greenhouse–Geisser correction was applied to ANOVA results. Additionally, data were checked for normality and homoscedasticity; when data were found to violate these assumptions, *post hoc* testing was performed by Kruskal–Wallis tests, as noted in the statistical tables. Otherwise, *post hoc* analyses were conducted according to the Tukey test. Complete analyses are presented in Tables 2–Tables 6.

**Table 2:** Statistical analysis of behavioral changes after 0, 4, or 8 weeks of UCMS in male and female mice

Two-way ANOVA	UCMS	Sex	UCMS × sex
EPM			
Total distance		*F*_(1,59)_ = 5.741 *p* = 0.019 (F > M)	*F*_(2,59)_ = 12.35 *p* < 0.0001
Time in open arms	*F*_(2,59)_ = 19.32 *p* < 0.0001	*F*_(1,59)_ = 6.477 *p* = 0.0136 (F > M)	*F*_(2,59)_ = 4.976 *p* = 0.010
Entries in open arms	*F*_(2,59)_ = 15.72 *p* < 0.0001	*F*_(1,59)_ = 6.924 *p* = 0.0108 (F > M)	
Distance in open arms	*F*_(2,59)_ = 16.25 *p* < 0.0001	*F*_(1,59)_ = 21.10 *p* < 0.0001 (F > M)	*F*_(2,59)_ = 3.665 *p* = 0.0316
OF			
Total distance	*F*_(2,80)_ = 3.241 *p* = 0.0443		
Time in center	*F*_(2,80)_ = 11.07 *p* < 0.0001		
Entries in center	*F*_(2,80)_ = 6.674 *p* = 0.0021		
Thigmotaxis ratio	*F*_(2,80)_ = 5.261 *p* = 0.0071		
CPA			
Post-pre time in LiCl side	*F*_(2,67)_ = 3.778 *p* = 0.0279	*F*_(1,67)_ = 4.299 *p* = 0.0420 (F > M)	*F*_(2,67)_ = 3.479 *p* = 0.0365
Marble burying			
No. of marbles buried	*F*_(2,40)_ = 3.972 *p* = 0.0267		*F*_(2,40)_ = 3.948 *p* = 0.0272
Splash test			
Time spent grooming	*F*_(2,40)_ = 4.496 *p* = 0.0177	*F*_(2,40)_ = 7.946 *p* = 0.0076 (F > M)	

F, Female; M, male.

**Table 3:** Statistical analysis of immunohistochemistry analysis of expression of FosB in prefrontal PV^+^ neurons after 0, 4, or 8 weeks of UCMS in male and female mice

Two-way ANOVA	UCMS	Sex	UCMS × sex
Percentage PV/FosB whole PFC	*F*_(2,18)_ = 22.45 *p* < 0.0001	*F*_(1,18)_ = 15.17 *p* = 0.0012	*F*_(2,18)_ = 3.327 *p* = 0.0603
Percentage PV/FosB PrL	*F*_(2, 218)_ = 9.711 *p* = 0.0015	*F*_(1,18)_ = 8.676 *p* = 0.0090	*F*_(2,18)_ = 2.982 *p* = 0.0776
Percentage PV/FosB IL	*F*_(2,18)_ = 18.51 *p* < 0.0001	*F*_(1,18)_ = 9.248 *p* = 0.0070	

**Table 4:** Statistical analysis of behavioral changes after 0 or 4 weeks of UCMS with or without chronic DREADD activation of prefrontal PV^+^ neurons in male mice

Two-way ANOVA	UCMS	DREADD	UCMS × DREADD
EPM			
Time in open arms	*F*_(1,32)_ = 6.831 *p* = 0.013		*F*_(1,32)_ = 5.004 *p* = 0.032
Entries in open arms			
Distance in open arms	*F*_(1,32)_ = 9.081 *p* = 0.005	*F*_(1,32)_ = 12.40 *p* = 0.001	
Total distance			
OF			
Time in center	*F*_(1,32)_ = 7.282 *p* = 0.011		
Entries in center		*F*_(1,32)_ = 4.154 *p* = 0.049	
Thigmotaxis ratio	*F*_(1,32)_ = 5.585 *p* = 0.024		
Total distance	*F*_(1,32)_ = 11.47 *p* = 0.002	*F*_(1,32)_ = 6.143 *p* = 0.019	
CPA			
Post-Pre time in LiCl side	*F*_(1,26)_ = 5.220 *p* = 0.031	*F*_(1,26)_ = 5.039 *p* = 0.033	
Marble burying			
No. of marbles buried	*F*_(1,32)_ = 9.285 *p* = 0.005		*F*_(1,32)_ = 3.227 *p* = 0.081
Splash test			
Time grooming	*F*_(1,26)_ = 7.465 *p* = 0.011		*F*_(1,26)_ = 3.609 *p* = 0.068

**Table 5:** Statistical analyses of AP firing after 0, 4, or 8 weeks of UCMS in IL layer II/III PV^+^ neurons from male and female mice

Test	UCMS	Sex	UCMS × sex	Stimulus	Stimulus × UCMS	Stimulus × sex	Stimulus × sex × UCMS
AP firing: sex as a factor							
Three-way ANOVA with repeated measures	*F*_(2,87)_ = 7.71, *p* < 0.001	*F*_(1,87)_ = 2.20, *p* = 0.14	*F*_(2,87)_ = 2.68, *p* = 0.074	*F*_(1.84,159.6)_ = 41.47, *p* < 0.001	*F*_(3.67,159.6)_ = 9.63, *p* < 0.001	*F*_(1.84,159.6)_ = 0.65, *p* = 0.51	*F*_(3.67,159.6)_ =1.42, *p* = 0.23
AUC analysis, two-way ANOVA	*F*_(2,87)_ = 6.99, *p* = 0.0015	*F*_(1,87)_ = 2.41, *p* = 0.12	*F*_(2,87)_ = 2.64, *p* = 0.077				
AP Firing by sex							
Males, two-way ANOVA	*F*_(2,39)_ = 10.86, *p* = 0.0002			*F*_(1.75,68.2)_ = 12.34, *p* < 0.0001	*F*_(14,273)_ = 3.88, *p* < 0.0001		
Females, two-way ANOVA	*F*_(2,48)_ = 1.27, *p* = 0.29			*F*_(1.91,91.9)_ = 35.67, *p* < 0.0001	*F*_(14,336)_ = 8.89, *p* < 0.0001		
AP height at threshold							
Three-way ANOVA with repeated measures	*F*_(2,87)_ = 4.99, *p* = 0.009	*F*_(1,87)_ = 0.93, *p* = 0.34	*F*_(2,87)_ = 0.008, *p* = 0.99	*F*_(1.05,91.4)_ = 25.05, *p* < 0.001	*F*_(2.10,91.4)_ = 0.18, *p* = 0.85	*F*_(1.05,91.4)_ = 1.71, *p* = 0.19	*F*_(2.10,91.4)_ = 0.51, *p* = 0.61
AP height at threshold by sex							
Males, two-way ANOVA	*F*_(2,39)_ = 2.11, *p* = 0.14			*F*_(1.12,43.8)_ = 14.15, *p* = 0.0003	*F*_(4,78)_ = 1.27, *p* = 0.29		
Females, two-way ANOVA	*F*_(2,48)_ = 3.03, *p* = 0.058			*F*_(1.03,49.5)_ = 15.30, *p* = 0.0002	*F*_(4,96)_ = 0.089, *p* = 0.99		
AP height at suprathreshold							
Three-way ANOVA with repeated measures	*F*_(2,87)_ = 20.04, *p* < 0.001	*F*_(1,89)_ = 0.04, *p* = 0.85	*F*_(2,89)_ = 0.97, *p* = 0.38	*F*_(1.16,101.0)_ = 254.23, *p* < 0.001	*F*_(2.32,101.0)_ = 28.87, *p* < 0.001	*F*_(1.16,101.0)_ = 1.19, *p* = 0.29	*F*_(2.32,101.1)_ = 1.53, *p* = 0.22
AP height at suprathreshold by sex							
Males, two-way ANOVA	*F*_(2,39)_ = 7.51, *p* = 0.0017			*F*_(1.05,40.8)_ = 141.1, *p* < 0.0001	*F*_(4,78)_ = 13.28, *p* < 0.0001		
Females, two-way ANOVA	*F*_(2,48)_ = 20.92, *p* < 0.0001			*F*_(1.21,58.1)_ = 131.1, *p* < 0.0001	*F*_(4,96)_ = 18.12, *p* < 0.0001		

Greenhouse–Geisser corrected for sphericity violations, as needed, indicated by noninteger degrees of freedom. Stimulus indicates intensity of current injected for AP firing and spike number for AP height.

**Table 6:** Statistical analyses of cell membrane and action potential properties after 0, 4, or 8 weeks of UCMS in IL layer II/III PV^+^ neurons from male and female mice

Property	UCMS	Sex	UCMS × sex	Effect
Resting membrane potential	*F*_(2,87)_ = 6.10 *p* = 0.0033	*F*_(1,87)_ = 2.10, *p* = 0.15	*F*_(2,87)_ = 2.37, *p* = 0.10	UCMS8 > control
Input resistance	*F*_(2,87)_ = 4.12 *p* = 0.020	*F*_(1,87)_ = 0.12, *p* = 0.73	*F*_(2,87)_ = 0.53, *p* = 0.59	UCMS4 > control
Total APs	*F*_(2,87)_ = 6.06, *p* = 0.0034	*F*_(1,87)_ = 2.86, *p* = 0.094	*F*_(2,87)_ = 1.61 *p* = 0.21	UCMS8 > control
First evoked AP amplitude (threshold)	*F*_(2,87)_ = 6.60 *p* = 0.0021	*F*_(1,87)_ = 1.11 *p* = 0.29	*F*_(2,87)_ = 0.16 *p* = 0.85	Control > UCMS8
Third evoked AP amplitude (threshold)	*F*_(2,87)_ = 6.63 *p* = 0.0021	*F*_(1,87)_ = 0.22 *p* = 0.64	*F*_(2,87)_ = 0.079 *p* = 0.92	Control > UCMS8
Third evoked AP amplitude (suprathreshold)	*F*_(2,87)_ = 39.32 *p* < 0.0001	*F*_(1,87)_ = 0.61 *p* = 0.44	*F*_(2,87)_ = 1.57 *p* = 0.21	Control > UCMS4 > UCMS8
Maximum frequency at rheobase	*F*_(2,87)_ = 2.86 *p* = 0.063	*F*_(1,87)_ = 2.08, *p* = 0.15	*F*_(2,87)_ = 2.29 *p* = 0.11	
Maximum frequency	*F*_(2,87)_ = 7.74 *p* = 0.0008	*F*_(1,87)_ = 23.76, *p* = 0.056	*F*_(2,87)_ = 3.97 *p* = 0.023	Control, UCMS4 > UCMS8
AP threshold	*F*_(2,87)_ = 2.51 *p* = 0.087	*F*_(1,87)_ = 1.68, *p* = 0.20	*F*_(2,87)_ = 1.38, *p* = 0.26	
AP half-width*	*F*_(2,87)_ = 1.29 *p* = 0.28	*F*_(1,87)_ = 0.52, *p* = 0.47	*F*_(2,87)_ = 3.48 *p* = 0.035	
Females only, Kruskal–Wallis test	H_(3)_ = 0.052, *p* = 0.97			
Males only, Kruskal–Wallis test	H_(3)_ = 11.21, *p* = 0.0037			UCMS8 > control

These tests were followed up by separate analyses by sex via Kruskal–Wallis tests. Comparisons in the Effect column are *post hoc* Tukey’s tests for parametric and Dunn’s multiple-comparisons tests for nonparametric statistics.

*Two-way ANOVA results are shown as non-normally distributed data, which could not be adjusted by mathematical transformation.

## Results

### Duration of UCMS required to alter behaviors and PV^+^ activation differs by sex

**Figure 1.**
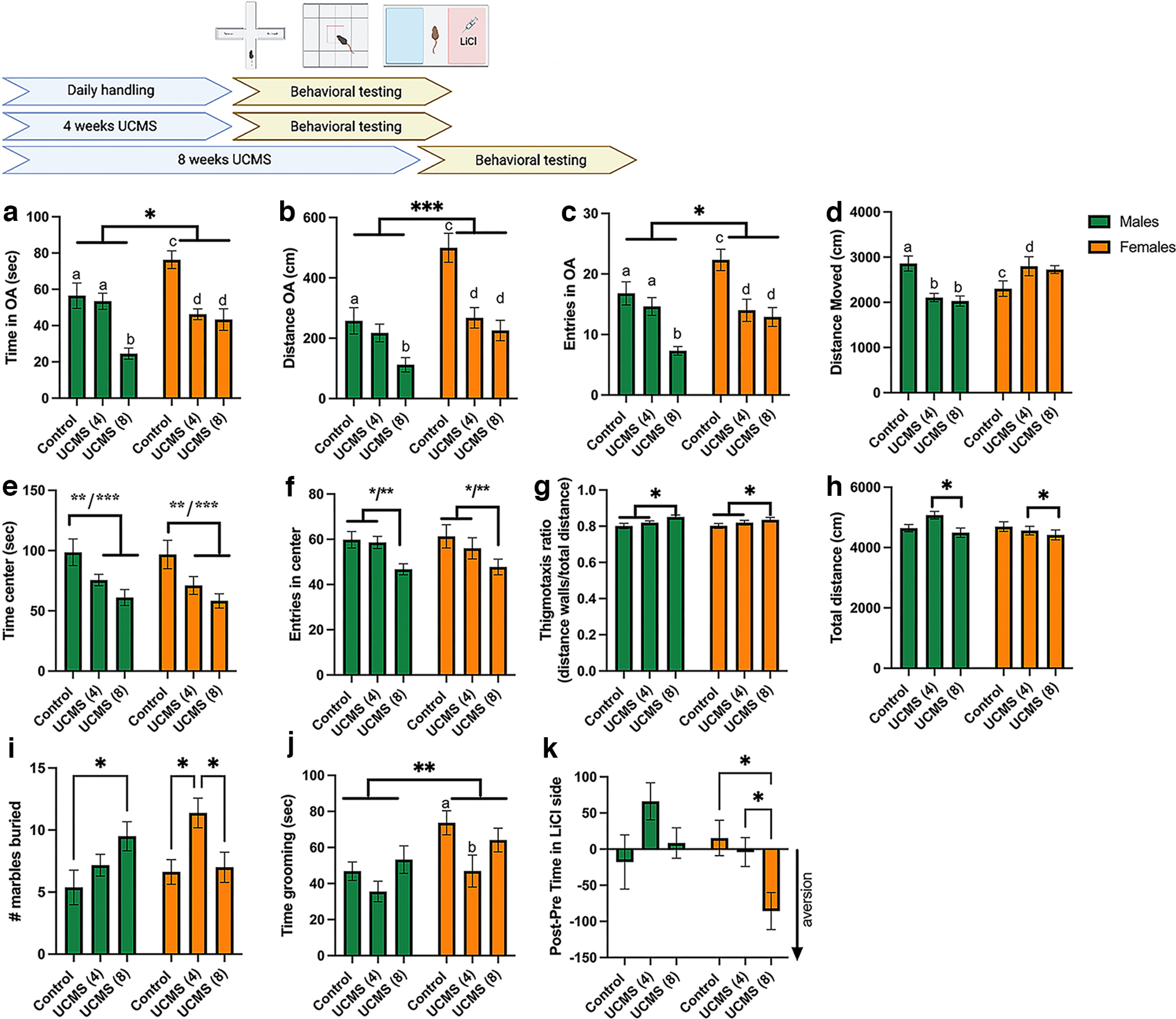
Effects of 4 and 8 weeks of UCMS on anxiety-like and depressive-like behaviors in male and female mice. Top, Schematic representation of experimental design. ***a–c***, In the EPM test, the time spent in the open arms (***a***), the distance traveled in the open arms (***b***), and the number of entries into the open arms (***c***) were significantly decreased after 4 and 8 weeks of UCMS in females (time in open arms: c > d, *p = *0.0005 and *p = *0.0001, respectively; distance in open arms: c > d, *p = *0.0002 and *p < *0.0001, respectively; entries into open arms: c > d, *p = *0.0030 and *p = *0.0007, respectively), while in males, only 8 weeks of UCMS decreased those end points when compared with controls (Cs) and males experiencing 4 weeks of UCMS (time in open arms: a > b, *p = *0.0001 and *p = *0.0001, respectively; distance in open arms: a > b, *p = *0.0187; entries into open arms: a > b, *p = *0.003). Altogether, females spent more time (*p = 0.0136), traveled a longer distance (***p < 0001), and made more entries (*p = 0.0108) in the open arms. ***d***, Total distance moved was decreased in males after 4 and 8 weeks of UCMS (a > b, *p = *0.0004) but tended to be increased in females after 4 weeks of UCMS (c < d, *p = *0.056). *N* = 9–16 mice/group [female: c, *N* = 9; UCMS (4), *N* = 10; UCMS (8), *N* = 10; male: c, *N* = 10; UCMS (4), *N* = 16; UCMS (8), *N* = 10]. ***e–h***, In the OF test, males and females were equally affected by 4 and 8 weeks of UCMS, as shown by decreased time spent in the center [***e***; C vs UCMS (4 weeks), **p = 0.0078; C vs UCMS (8 weeks), ***p < 0.0001], decreased the number of entries in the center [***f***; C vs UCMS (8 weeks), **p = 0.0031, UCMS (4 weeks) vs UCMS (8 weeks), *p = 0.0163], increased the thigmotaxis ratio [***g***; C vs UCMS (8 weeks), **p = 0.0055] and changes in overall activity [***h***; UCMS (4 weeks) vs UCMS (8 weeks), *p = 0.0343]. *N* = 10–20 mice/group [female: C, *N* = 13; UCMS (4 weeks), *N* = 14; UCMS (8 weeks), *N* = 14; male: C, *N* = 10; UCMS (4 weeks), *N* = 20; UCMS (8 weeks), *N* = 15]. ***i***, Marble burying. Females exposed to 4 weeks of UCMS and males exposed to 8 weeks of UCMS buried more marbles (females: C vs UCMS (4 weeks), *p = 0.0159; UCMS (4 weeks) vs UCMS (8 weeks), *p = 0.0281; males: C vs UCMS (8 weeks), *p = 0.0404). *N* = 7–8/group [females: C, *N* = 8; UCMS (4 weeks), *N* = 8; UCMS (8 weeks), *N* = 8; male: C, *N* = 7; UCMS (4 weeks), *N* = 7; UCMS (8 weeks), *N* = 8]. ***j***, Splash test. In the splash test, females groomed more than males (**p = 0.0076). Females appeared more sensitive to the effects of UCMS, as 4 weeks of UCMS significantly decreased the time spent grooming (a vs b, *p = *0.0213). *N* = 7–8/group [females: c, *N* = 8; UCMS (4 weeks), *N* = 8; UCMS (8 weeks), *N* = 8; male: c, *N* = 7; UCMS (4 weeks), *N* = 7; UCMS (8 weeks), *N* = 8]. ***k***, In the CPA test, only females showed increased aversion toward a low dose of LiCl after 8 weeks of UCMS (vs C, *p = 0.0106; vs UCMS (4 weeks), *p = 0.0477). *N* = 8–15 mice/group [females: c, *N* = 14; UCMS (4 weeks), *N* = 14; UCMS (8 weeks), *N* = 13; males: c, *N* = 9; UCMS (4 weeks), *N* = 8; UCMS (8 weeks), *N* = 15]. Data are presented as the mean ± SEM.

**Figure 2.**
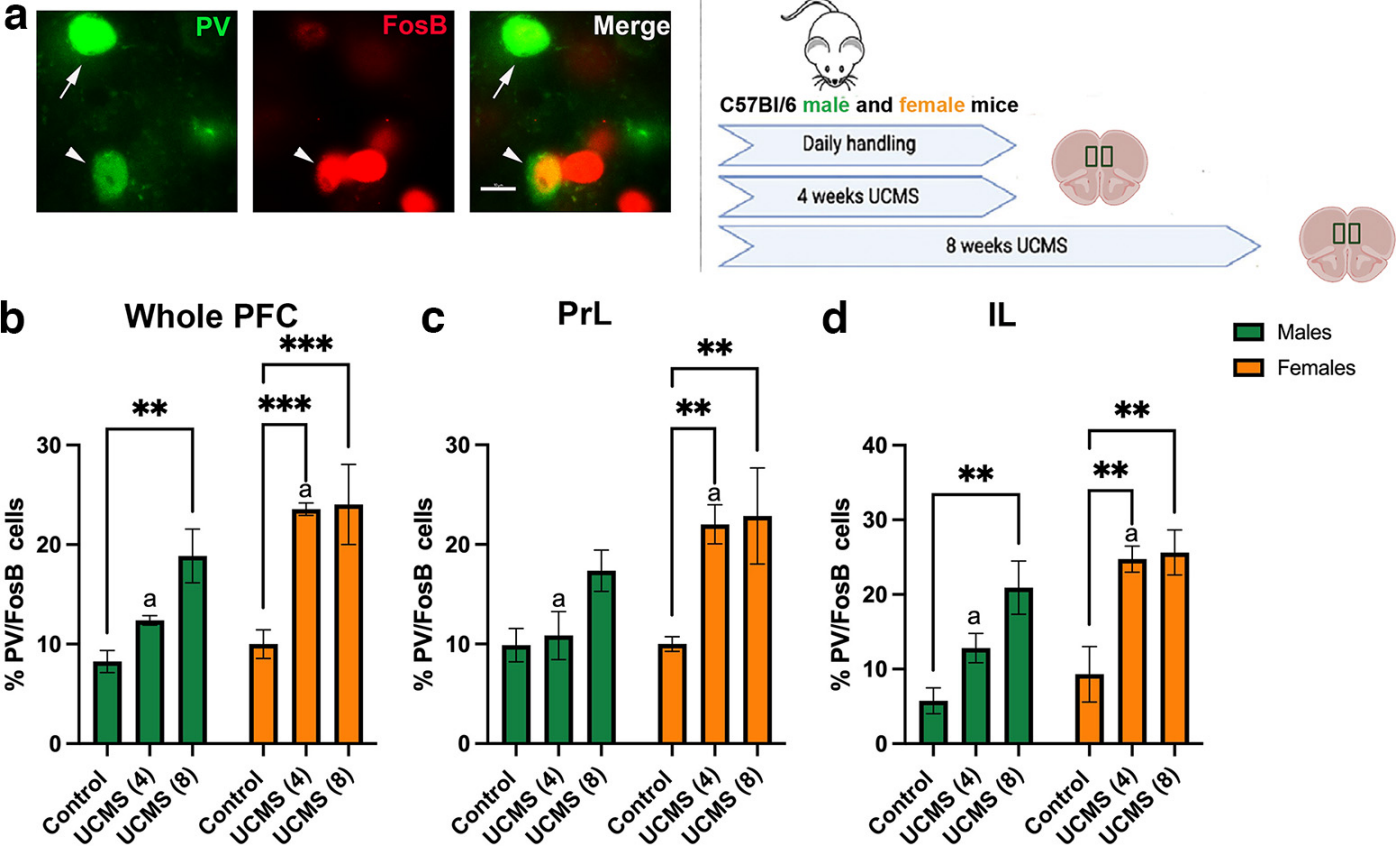
Cellular changes in female and male prefrontal PV^+^ neurons after 4 and 8 weeks of UCMS are indicative of sex-specific patterns of stress-induced increases in their activity. ***a***, Left, Representative picture of immunofluorescent signal of PV (green), ΔFosB (red), and their colocalization in the PFC of mice. Right, Experimental timeline. ***b–d***, PV/ΔFosB immunohistochemistry analyses. ***b***, Increased percentage of prefrontal PV^+^ neurons expressing the marker of chronic activity, ΔFosB, in the whole mPFC after 4 and 8 weeks of UCMS in females [UCMS (4 weeks) vs controls, ***p = 0.0002; UCMS (8 weeks) vs controls, ***p = 0.0003], and after 8 weeks of UCMS in males [UCMS (8 weeks) vs controls, **p = 0.0022]. ***c***, ***d***, This is paralleled by subregion analyses wherein the PrL (***c***) UCMS did not change the percentage of PV^+^ neurons expressing ΔFosB in males but increased it in females [control vs UCMS (8 weeks) **p = 0.0046], and in the IL (***d***) 8 weeks of UCMS significantly increased percentage of PV^+^ neurons expressing ΔFosB in both males and females (males, **p = 0.0014; females, **p = 0.0024), while 4 weeks of UCMS increased this number in females (**p = 0.0020). We also observed significant sex differences, particularly after 4 weeks of UCMS, when females displayed more PV neurons expressing FosB than males in the whole PFC, PrL region, and IL region (whole PFC, ^a^*p = *0.0015; PrL, ^a^*p = *0.0085; IL, ^a^*p = *0.0167). *N* = 4–5/group [female: c, *N* = 4; UCMS (4 weeks), *N* = 4; UCMS (8 weeks), *N* = 4; male: c, *N* = 5; UCMS (4 weeks), *N* = 4; UCMS (8 weeks), *N* = 4]. Data are presented as the mean ± SEM.

**Figure 3.**
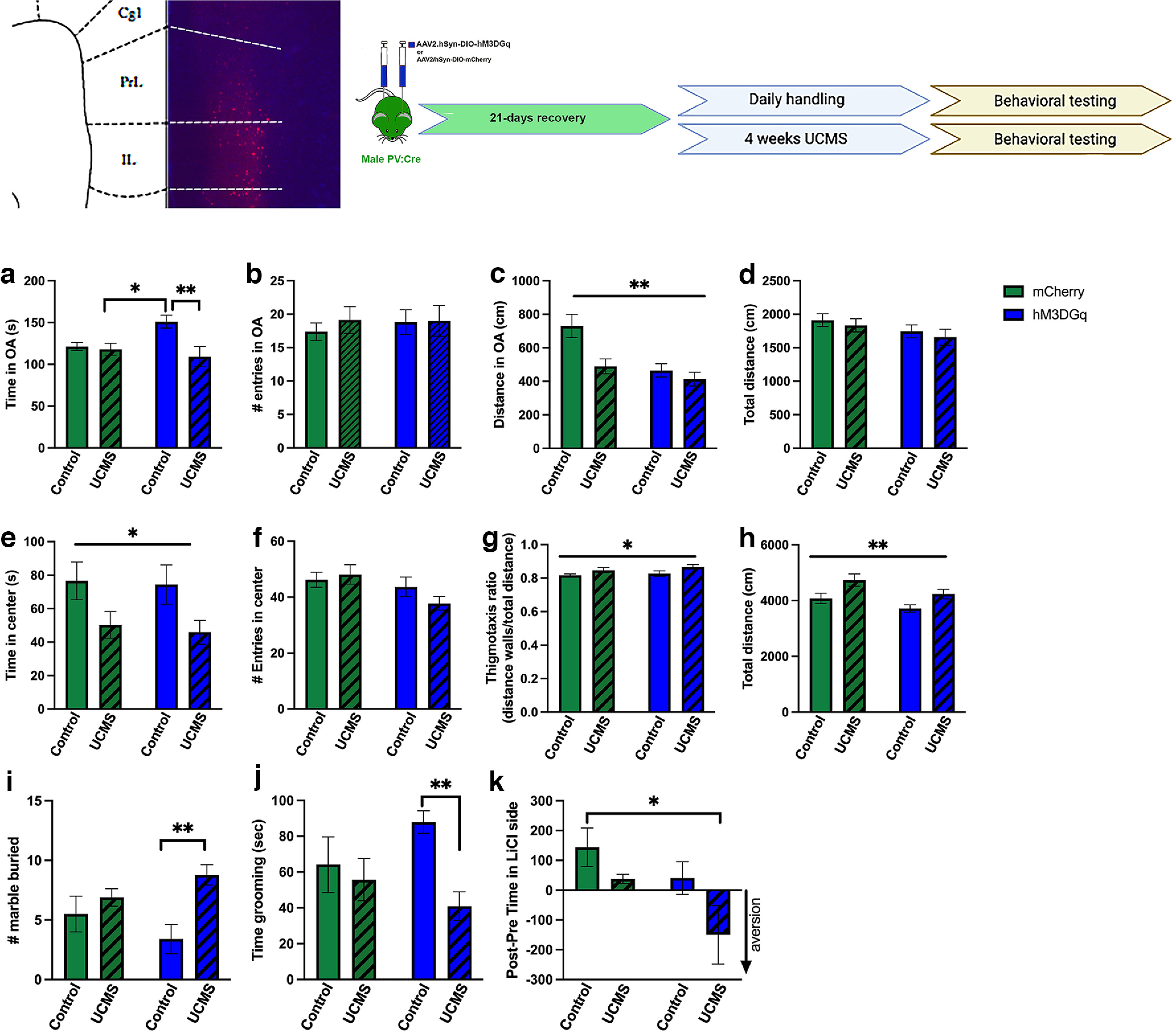
Activation of prefrontal PV^+^ neurons in male mice affects the expression of anxiety-like and depressive-like behaviors under baseline and stressful conditions. Top left, Representative image of mCherry-DREADD virus expression in the PrL and IL regions of the PFC. Top right, Schematic representation of experimental design. ***a–d***, In the EPM test, UCMS interacted with the chronic activation of prefrontal PV^+^ neurons to modulate the time spent in the open arms (***a***; *p = 0.0441; **p = 0.0052), but no other significant interactions were observed. ***e*–*h***, In the OF test, UCMS decreased time in the center (***e***; *p = 0.011), increased thigmotaxis ratio (***g***; *p = 0.0244), and increased overall activity (***h***; **p = 0.0019). ***i***, ***j***, In the marble-burying test, a significant UCMS effect was driven by a higher number of marbles buried by UCMS/hM3DGq mice compared with their nonstressed controls (**p = 0.0038; ***i***); a similar effect was observed in the splash test (***j***), with decreased time spent grooming by UCMS/hM3DGq mice compared with their nonstressed controls (**p = 0.0088). ***k***, In the CPA test, significant effects of stress and virus were detected; Tukey’s *post hoc* analyses revealed that the aversion score of mice from the UCMS/hM3DGq was lower than that of control mice (*p = 0.021). *N* = 8–11/group (C/mCherry, *N* = 8; UCMS/mCherry, *N* = 11; C/hM3DGq, *N* = 8; UCMS/hM3DGq, *N* = 9). Data are presented as the mean ± SEM.

**Figure 4.**
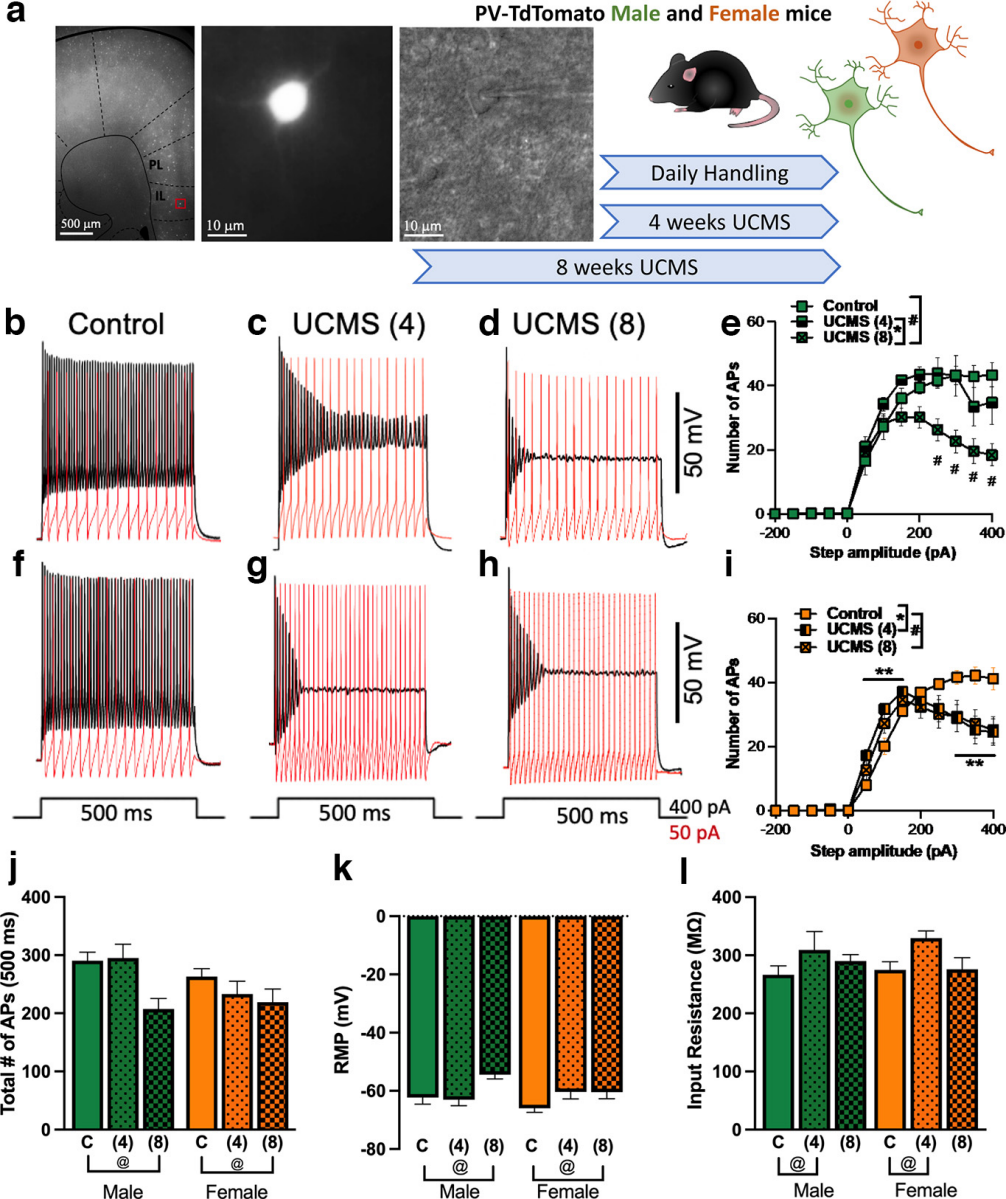
Altered neuronal excitability patterns of IL layer II/III PV^+^ neurons after 4 and 8 weeks of UCMS. ***a***, Representative images of recorded neurons (left, position in slice; center, fluorescence indicating PV presence; right, IR-DIC image of patched neuron) and schematic of experimental design. ***b–d***, Representative traces of PV^+^ neuron firing in male neurons showing the characteristic nonadapting firing patterns of PV^+^ neurons (control) at threshold (i.e., 50 pA, red) and suprathreshold (i.e., 400 pA, black) current injection (***b***) and altered firing patterns after 4 weeks [UCMS (4 weeks)] and 8 weeks [UCMS (8 weeks)] of UCMS (***c***, ***d***). ***e***, Male PV^+^ neurons showed no significant differences in the number of APs after either 4 or 8 weeks of UCMS at threshold current injections, but a significant reduction in the number of events at suprathreshold step currents (i.e., from 250 pA) after only 8 weeks of UCMS [**#***p *<* *0.001, control vs UCMS (8 weeks), Tukey’s *post hoc* test; exact *p* values: control vs UCMS (8 weeks): 250 pA, *p *=* *0.0017; 300 pA, *p *=* *0.0003; 350 pA, *p *=* *0.0003; 400 pA, *p *=* *0.0001]. ***f–h***, Representative traces of PV^+^ neuron firing in female neurons showing characteristic PV^+^ neuron firing patterns in controls (***f***), as above, and alterations after UCMS (4 weeks) and UCMS (8 weeks; ***g***, ***h***). ***i***, Female PV^+^ neurons showed maladaptive firing patterns with increased numbers of APs at lower current injections [*p <* *0.05, Control vs UCMS (4 weeks), Tukey’s *post hoc* test; exact *p* values: control vs UCMS (4 weeks): 50 pA, *p *=* *0.0016; 100 pA, *p *=* *0.0009; 150 pA, *p *=* *0.036], but a reduced number of events at higher, suprathreshold current injections [**p <* *0.001, control vs UCMS (4 weeks); **#***p *<* *0.001, control vs UCMS (8 weeks), Tukey’s *post hoc* tests; exact *p* values: control vs UCMS (4 weeks): 300 pA, *p *=* *0.036; 350 pA, *p *=* *0.0059; 400 pA, *p *=* *0.012; control vs UCMS (8 weeks): 350 pA, *p *=* *0.021; 400 pA, *p *=* *0.017]. ***j***, Total number of APs fired across the 500 ms stimulus duration for all stimulus intensities decreased after 8 weeks of UCMS, regardless of sex [@*p *<* *0.05, Control vs UCMS (8 weeks), Tukey’s *post hoc* test for main effect of UCMS; exact *p* value: *p *=* *0.0090]. ***k***, RMP was significantly increased after UCMS (8 weeks), independent of sex [@*p *<* *0.05, control vs UCMS (8 weeks), Tukey’s *post hoc* test for main effect of UCMS, exact *p *=* *0.0080]. ***l***, Input resistance was significantly increased after UCMS (4 weeks), independent of sex [@*p *<* *0.05, control vs UCMS (4 weeks), Tukey’s *post hoc* test for main effect of UCMS; exact *p *=* *0.015]. Data in histograms are presented as the mean ± SEM for control (C), 4 weeks of UCMS (4 weeks) and 8 weeks of UCMS (8 weeks) by sex (male, green; female, orange). *N* = 4–6 mice/group, *n* = 11–19 recorded cells/group [females: C: *N* = 6, *n* = 19; 4 weeks: *N* = 5, *n* = 17; 8 weeks: *N* = 5, *n* = 15; males: C: *N* = 4, *n* = 14; 4 weeks: *N* = 4, *n* = 11; 8 weeks: *N* = 5, *n* = 17). Individual mice contributed 2–6 cells to the experiment (mean *n* per *N*, 3.23; median *n* per *N*, 3).

**Figure 5.**
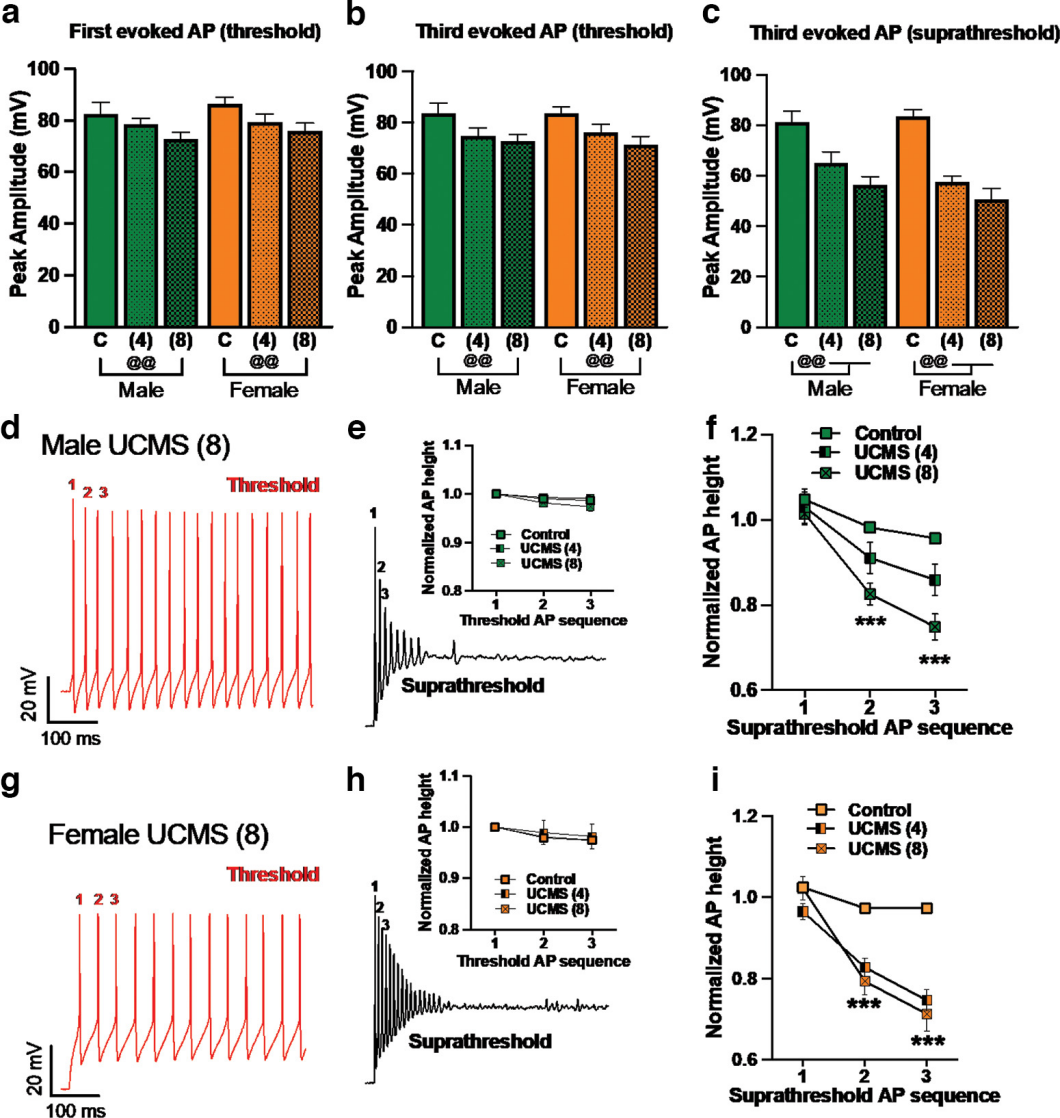
UCMS reduced AP height as a feature of the adaptation of IL layer II/III PV^+^ neurons. Male and female PV-tdTomato mice were exposed to handling [control (C)], 4 weeks of UCMS (4 weeks), or 8 weeks of UCMS (8 weeks); and 24 h later, IL layer II/III PV^+^ neurons were selected for recording based on the presence of red fluorescence. AP magnitudes were assessed for the first 3 spikes after current onset at threshold (50 pA) and suprathreshold (400 pA) current intensities. ***a***, The amplitude of the first AP was reduced after 8 weeks of UCMS, regardless of sex [@@*p *<* *0.01, control vs UCMS (8 weeks), Tukey’s *post hoc* test for main effect of UCMS; exact *p *=* *0.0014]. ***b***, The height of the third evoked AP at threshold current intensity was reduced after 8 weeks of UCMS, regardless of sex [@@*p *<* *0.01, control vs UCMS (8 weeks), Tukey’s *post hoc* test for main effect of UCMS; exact *p *=* *0.0015]. ***c***, The amplitude of the third AP evoked by suprathreshold stimuli was reduced after both 4 and 8 weeks of UCMS, regardless of sex [@@*p *<* *0.01, control vs UCMS (4 weeks) and UCMS (8 weeks), Tukey’s *post hoc* test for main effect of UCMS; exact *p *<* *0.0001, UCMS (4 weeks); *p *<* *0.0001, UCMS (8 weeks)], with effects for 4 weeks but not 8 weeks of UCMS largely driven by females despite lack of UCMS by sex interaction [control vs UCMS (4 weeks): females, *p *<* *0.0001; males, *p *=* *0.011; Control vs UCSM (8 weeks): females, *p *<* *0.0001; males, *p *<* *0.0001). ***d***, Representative traces illustrating the 3 APs measured at threshold (left, red trace) and suprathreshold (right, black trace) current intensities in neurons from male mice. ***e***, In male mice, AP heights were progressively shorter from AP1 to AP3 (Tukey’s test exact *p* values: AP1 vs AP2, *p *=* *0.015; AP1 vs AP3, *p *=* *0.0003; AP2 vs AP3, *p *=* *0.0021). UCMS did not significantly impact AP height. Data are displayed with AP heights normalized to threshold AP1 height. ***f***, AP height, expressed as a ratio to the first AP generated under threshold current stimulation, was significantly reduced after UCMS (8 weeks) in male mice [control vs UCMS (8 weeks): AP1, *p *=* *0.080; AP2, *p *=* *0.0014; AP3, *p *=* *0.0003]. ***g***, Representative traces illustrating APs measured at threshold (red trace) and suprathreshold (black trace) current intensities in neurons from female mice. ***h***, In females, UCMS did not significantly impact AP heights at threshold, with data displayed normalized to the height of the first AP, although AP height did decrease with each spike (Tukey’s test exact *p* values: AP1 vs AP2, *p *=* *0.0022; AP1 vs AP3, *p *=* *0.0002; AP2 vs AP3, *p *<* *0.0001). ***i***, At suprathreshold current intensity, AP height was decreased relative to control following both UCMS (4 weeks: AP1, *p *=* *0.0084; AP2, *p *<* *0.0001; AP3, *p *<* *0.0001) and UCMS (8 weeks: AP1, *p *=* *0.022; AP2, *p *<* *0.0001; AP3, *p *<* *0.0001), without differences between UCMS (4 weeks) and UCMS (8 weeks), in female mice. Data are presented as the mean ± SEM for control (C), 4 weeks of UCMS (4 weeks), and 8 weeks of UCMS (8 weeks) by sex (male, green; female, orange). *N* = 4–6 mice/group, *n* = 11–19 recorded cells/group [females: C: *N* = 6, *n* = 19; 4 weeks: *N* = 5, *n* = 17; 8 weeks: *N* = 5, *n* = 15; males: C: *N* = 4, *n* = 14; 4 weeks: *N* = 4, *n* = 11; 8 weeks: *N* = 5, *n* = 17]. Individual mice contributed 2–6 cells to the experiment (mean *n* per *N*, 3.23; median *n* per *N*, 3).

Previous work has indicated differential vulnerability to UCMS by sex: female mice display increased anxiety-like behaviors, increased mPFC PV mRNA, and a greater proportion of PV^+^ neurons in the mPFC expressing c-Fos after 4 weeks of exposure to UCMS (Shepard et al., 2016; Page et al., 2019), whereas an 8 week exposure to UCMS is required to induce a similar anxiety-like phenotype in male mice (Monteiro et al., 2015). Here, these two UCMS exposures were compared directly to examine the relationship among sex, changes in prefrontal PV^+^ neuron activity, and vulnerability to stress-related anxiety-like and depressive-like phenotypes. We predicted that in females, 4 weeks of UCMS would be sufficient not only to heighten anxiety-like behaviors, but also to increase activation of prefrontal PV^+^ neurons. Conversely, we postulated that the activation of male prefrontal PV^+^ neurons and anxiety-like behaviors would be resilient to change after 4 weeks, but not 8 weeks, of UCMS. For all studies, main findings are described below and in Figures 1–5, and complete statistics are presented in Tables 2–6.

To examine sex differences in susceptibility to UCMS load altering anxiety-like behaviors, mice were exposed to EPM, OF, and marble-burying tests after exposure to 4 or 8 weeks of UCMS or handling control. We also conducted a splash test and a CPA test to assess changes in other behavioral domains relevant to a depressive-like phenotype.

In the EPM, significant sex by UCMS group interactions were found for time spent in the open arms and distance traveled in the open arms (Table 2). Tukey’s *post hoc* tests showed that females exposed to 4 and 8 weeks of UCMS spent less time in the open arms compared with controls (*p *<* *0.001 and *p *<* *0.0001, respectively; Fig. 1*a*, orange bars). In males, 8 weeks of UCMS were needed to reduce the time spent in the open arms (*p *<* *0.0001; Fig. 1*a*, green bars). Similar results were found for the distance traveled in the open arms (Fig. 1*b*, Table 1). Regarding the number of entries into the open arms, the main effects of sex and UCMS group were found (Table 2). Tukey’s *post hoc* tests showed that 4 and 8 weeks of UCMS decreased the number of entries in females (*p *=* *0.003 and *p *=* *0.0007, respectively; Fig. 1*c*). In males, only 8 weeks of UCMS induced a significant decrease in the number of entries (vs controls, *p *=* *0.0005; vs 4 weeks of UCMS, *p *=* *0.003; Fig. 1*c*). Those behavioral changes in the EPM are suggestive of enhanced anxiety-related behaviors after 4 and 8 weeks of UCMS in females, but only after 8 weeks of UCMS in males. Finally, the significant interaction between sex and UCMS group for total distance traveled showed that UCMS differently affected locomotor activity of males and females in the maze: UCMS (4 and 8 weeks) decreased the activity of males (*p *=* *0.0004), while 4 weeks of UCMS tended to increase the activity of females (*p *=* *0.056; Fig. 1*d*). In the OF test (OFT), significant effects of the UCMS group were found, without sex effects or interactions (Table 2). Four and 8 weeks of UCMS decreased the time spent in the center (*p *=* *0.078 and *p *<* *0.0001, respectively; Fig. 1*e*), while 8 weeks of UCMS decreased the number of entries into the center (vs controls, *p *=* *0.003; vs 4 weeks of UCMS, *p *=* *0.016; Fig. 1*f*) and increased the thigmotaxis ratio (vs controls, *p *=* *0.0055; Fig. 1*g*), all indicative of increased anxiety-like behaviors in both sexes. We also noted a decrease in the total distance traveled after 8 weeks of UCMS in both males and females (vs 4 weeks of UCMS, *p *=* *0.034; Fig. 1*h*).

In the marble-burying test, a main effect of UCMS was found as well as a significant interaction between sex and UCMS (Fig. 1*i*, Table 2). *Post hoc* analyses revealed that 4 weeks of UCMS increased the number of marbles buried by females (*p = *0.0159 vs controls; *p = *0.0281 vs 8 weeks of UCMS); a similar increase was observed in males only after 8 weeks of UCMS (*p = *0.0404 vs controls), suggesting increased anxiety-like behaviors after 4 weeks of UCMS in females and after 8 weeks of UCMS in males. Unexpectedly, there was no significant increase in the number of marbles buried by females exposed to 8 weeks of UCMS.

In the splash test, the time spent grooming was affected by UCMS and sex (Fig. 1*j*, Table 2). Females overall groomed more than males, and 4 weeks of UCMS decreased the time spent grooming (*p = *0.0261), an effect driven by females (*p = *0.0213; males, *p > *0.1) that could indicate enhanced depressive-like behaviors.

Finally, in the CPA test, significant UCMS group, stress, and sex by UCMS group interactions were observed (Table 2). In males, no significant effect of UCMS (4 or 8 weeks) was observed. In females, 8 weeks of UCMS increased aversion to a low dose of LiCl (vs controls, *p *=* *0.011; vs 4 weeks of UCMS, *p *=* *0.047; Fig. 1*k*). One-sample *t* tests further showed that females with 8 weeks of UCMS had an aversion score significantly <0, indicating aversion (*t*_(12)_ = 3.356; *p = *0.006), while the aversion score of males with 4 weeks of UCMS was significantly above chance level (*t*_(7)_ = 2.587; *p = *0.0361), which could suggest a preference for the LiCl-paired side. However, we further observed that overall locomotor activity in the testing arena significantly decreased between the pretest phase and the test phase in males with 4 weeks of UCMS only (UCMS groups by test phase interaction: *F*_(2,30)_ = 11.92; *p=* 0.0002; *post hoc* analyses for the 4 week UCMS group: *p* < 0.0001; data not shown). In this group, the distance traveled is reduced mostly on the LiCl-associated side (*p = *0.060 for comparison between pre-test and test) but not on the saline-associated side (*p > *0.99). This observation, combined with the increased time spent in the LiCl-associated side during the test phase, could suggest that males exposed to 4 weeks of UCMS displayed immobility on the side of the arena associated with the aversive stimulus, which might indicate fear-like behavior.

Sex-specific changes in mPFC PV^+^ neuron activity after 4 and 8 weeks of UCMS were assessed by ΔFosB expression (Fig. 2), a molecular marker of chronic activity. We found significant UCMS and sex effects for the percentage of PV neurons expressing FosB counted throughout the mPFC (Fig. 2*b*, Table 3). In females, both 4 and 8 weeks of UCMS increased the percentage of PV cells expressing FosB, compared with controls (*p *=* *0.0002 and *p *=* *0.0003, respectively), while in males, only 8 weeks of UCMS increased the percentage of PV cells expressing FosB compared with controls (*p *=* *0.0022). These increases in PV/FosB cells also differed by sex in their spatial distribution within the mPFC. In PrL, the main effects of UCMS and sex, and a nearly significant interaction between sex and UCMS, were observed (Table 3). *Post hoc* analyses revealed that only females displayed an increased percentage of PV cells expressing FosB after 4 and 8 weeks of UCMS (*p *=* *0.0043 and *p *=* *0.0046; respectively; Fig. 2*c*). We also noticed that females exposed to 4 weeks of UCMS had more PV/FosB colabeled cells than males exposed to 4 weeks of UCMS (*p *=* *0.0085). In the IL area, significant main effects of sex and UCMS group were found, without interaction between the factors (Table 3), indicating that while the patterns differed between the sexes, both females and males showed IL adaptation. Specifically, females showed an increased percentage of PV cells expressing FosB after both 4 and 8 weeks of UCMS (*p *=* *0.0020 and *p *=* *0.0024, respectively; Fig. 2*d*). In males, an increased percentage of PV cells expressing FosB was observed only after 8 weeks of UCMS (*p *=* *0.0014; Fig. 2*d*). We also observed that the main effect of sex is driven mostly by levels of PV/FosB colabeling after 4 weeks of UCMS (females > males; *p = *0.0167), rather than by baseline sex differences or differences after prolonged exposure to UCMS.

### Activating prefrontal PV^+^ neurons in males under baseline and stressful conditions affects the expression of anxiety-like and depressive-like behaviors

The observation of increased expression of the chronic activity marker ΔFosB throughout the mPFC in both sexes after 8 weeks of UCMS but only in females after 4 weeks of UCMS suggested that the rather moderate changes in anxiety-like behaviors in males after a short (4 weeks) period of UCMS could be because of the resilience of their prefrontal PV^+^ cells to stress-induced adaptations. Thus, we hypothesized that increasing PV neuron activity during UCMS exposure in males would be sufficient to heighten stress-induced behavioral changes after 4 weeks of UCMS. PV:Cre male mice were injected into the mPFC (PrL and IL) with a Gq DREADD virus or an mCherry control virus and exposed to daily handling or UCMS for 4 weeks. CNO was injected intraperitoneally 30 min before daily handling or stress exposure. Twenty-four hours after the last manipulations, behaviors were tested in the EPM, OF, marble-burying, splash, and CPA tests (Fig. 3, Table 4). Previous work reported that chronic CNO treatment in adult male mice did not impact their overall behaviors (Page et al., 2019), so no vehicle group was included in the study.

Unfortunately, our data failed to provide convincing evidence for enhanced stress-induced anxiety as a response to 4 weeks of UCMS and activation of PV^+^ cells. First, while we were able to replicate the male behaviors observed in the open field in the first experiment in response to UCMS (enhanced anxiety-like behavior as shown by reduced time in the center of the arena and increased thigmotaxis ratio), this effect was independent of the DREADD virus (main UCMS effect: Fig. 3*e*, *p *=* *0.011; Fig. 3*g*, *p *=* *0.024).

In the assays for which we previously reported no change in response to 4 weeks of UCMS (EPM, marble-burying, and splash tests), we observed significant interactions between UCMS and the DREADD virus (Table 4). However, Tukey’s *post hoc* analyses revealed that mice expressing the Gq DREADD virus and handled daily (no stress exposure) spent more time in the open arms of the EPM compared with the two UCMS groups (vs mCherry/UCMS, *p *=* *0.044; vs Gq/UCMS, *p *=* *0.0052; Fig. 3*a*), buried fewer marbles than Gq/UCMS mice (*p *=* *0.004; Fig. 3*i*), and groomed more after being sprayed with a sucrose solution than Gq/UCMS mice (*p *=* *0.008; Fig. 3*j*), all suggestive of reduced anxiety-like and depressive-like behaviors. Other recorded behaviors were affected by both stress and virus, including distance traveled in the open arms of the EPM (Fig. 3*c*) and aversion score in the CPA (Fig. 3*k*), where we noted that Gq/UCMS mice had a higher aversion score for a low dose of LiCl (vs mCherry/controls, *p *=* *0.022). However, the significant difference in aversion score between mCherry/controls and Gq/UCMS mice is likely because of a combinatorial effect of a noted preference for the LiCl side in mCherry/control mice (though not significant per one-sample *t* test vs chance level, *p > 0.05*), and preference for the saline side in Gq-UCMS mice (though not significant per one-sample *t* test vs chance level, *p > 0.05*). The latter challenges the interpretation of results in Gq/UCMS mice. Others have reported that CNO alone has the potential to disrupt affective motivation (Derman et al., 2020), which could contribute to some of the effects observed in the CPA test.

### Changes in PV^+^ interneuron excitability in infralimbic cortex layers II/III align with sex differences in behavioral adaptation to UCMS

The pattern of UCMS-induced increase in ratios of FosB-labeled PV neurons in IL layers in males and females parallels the sex-specific temporal pattern by which UCMS behavioral effects emerged, suggesting that functional changes in IL neurons, as opposed to PrL neurons, may play an important role in the behavioral consequences of UCMS in both sexes. Additionally, previous studies in male rats demonstrated changes in PV^+^ neuron number and GABA regulation of pyramidal neuron activity in IL layers II/III after 9 weeks of chronic stress (Czéh et al., 2018). Thus, we set out to determine whether the differential time course by sex for the emergence of anxiety-like behavior following UCMS would be mirrored by functional changes in PV^+^ neurons in IL layers II/III (Fig. 4*a*, experimental schematic).

UCMS produced distinct changes in the evoked firing patterns of IL PV^+^ neurons. Analysis by three-way ANOVA showed between-subjects effects by UCMS condition (Table 5; control vs 8 weeks of UCMS, *p *=* *0.001; 4 weeks of UCMS vs 8 weeks of UCMS, *p *=* *0.027), while a sex by UCMS interaction emerged at trend level (*p *=* *0.074). We observed a similar trend for sex by UCMS interaction when data were transformed to the area under the curve of stimulus intensity versus AP firing (*p *=* *0.077). Because of the substantial differences in the timing of behavioral changes emerging by sex and heteroskedasticity of the data across stimulus intensities, we elected to analyze the relationship between current intensity and excitability separately by sex. Sex-specific two-way ANOVAs (Table 5) showed distinct alterations in neural firing patterns from male versus female mice following UCMS. PV^+^ neurons from male mice (Fig. 4*b–d*, representative traces) showed significant adaptation, characterized by an inability of neurons to maintain their firing rates across the entire 500 ms current duration at suprathreshold stimulus intensities, after 8 weeks, but not 4 weeks, of UCMS (Fig. 4*e*), compared with controls (Fig. 4, black traces, compare *b*, *d*). Conversely, female mice (Fig. 4*f–h*, representative traces) showed UCMS-induced changes at both the 4 and 8 week timepoints, with two specific components to these alterations (Fig. 4*i*). First, PV^+^ neurons became hyperexcitable after 4 weeks of UCMS, marked by increased numbers of APs at lower stimulus intensities (Fig. 4, red traces, compare *f* and *g*, *h*). Second, AP firing showed adaptation at suprathreshold stimulus intensities after both 4 and 8 weeks of UCMS, compared with controls (Fig. 4, black traces, compare *f* and *g*, *h*). The timing of the emergence of firing adaptations was in line with behavioral findings. This adaptation was most pronounced at 8 weeks in both sexes, reducing the total number of APs fired over 500 ms across all stimulus intensities (Fig. 4*j*; controls vs 8 weeks of UCMS, *p *=* *0.0090; 4 weeks of UCMS vs 8 weeks of UCMS, *p *=* *0.034). These altered spiking patterns were accompanied by changes in some basic neural membrane and firing properties (Table 6). RMP was elevated only after 8 weeks of UCMS in both sexes (Fig. 4*k*; controls vs 8 weeks of UCMS, *p *=* *0.0024), a neuronal property that indicates the membrane is depolarized relative to the control condition. Input resistance was elevated after 4 weeks, but not 8 weeks, of UCMS (Fig. 4*l*; controls vs 4 weeks of UCMS, *p *=* *0.018), suggestive of increased excitability.

Because adaptation can be characterized not only by early cessation of firing but also by changes in the amplitude of action potentials, the height of the first three AP spikes following the onset of current injection was quantified according to previous reports (Bean, 2007; Brickley et al., 2007). The heights of the first evoked AP (Fig. 5*a*) and the third evoked AP (Fig. 5*b*) at threshold (i.e., 50 mA current injection) were significantly reduced after 8 weeks of UCMS, relative to control (AP1 control vs 8 weeks of UCMS, *p *=* *0.0014; AP3 control vs 8 weeks of UCMS, *p *=* *0.0015; Table 6), regardless of sex. At suprathreshold current injections (i.e., 400 pA), where adaptation was observed, the third AP height was reduced after both 4 weeks (*p *<* *0.0001) and 8 weeks (*p *<* *0.0001) of UCMS, relative to control (Fig. 5*c*), independent of sex. Three-way ANOVAs comparing the effects of UCMS and sex on changes in AP height across the first three spikes showed minimal effects of UCMS or sex at threshold (Table 5), although AP height decreased across each successive spike. However, at suprathreshold current intensities, this progressive reduction in AP height differed in magnitude according to UCMS exposure. While the three-way interaction among AP number, UCMS, and sex was not significant for threshold or suprathreshold stimulus intensities (Table 5), planned comparisons within sex were performed in alignment with AP firing analyses. In males, no effect of UCMS was seen at threshold stimulation (Fig. 5*d*, red trace, *e*, quantification), but, when suprathreshold stimulation was administered (Fig. 5*d*, black trace, *f*, quantification), changes in AP height were impacted by UCMS: 8 week UCMS AP heights were significantly lower than those of controls on AP2 (*p *<* *0.0001) and AP3 (*p *<* *0.0001), whereas 4 weeks of UCMS yielded only a trending difference from controls on AP3 (*p *=* *0.067). As seen for spike number, adaptation of AP height in females developed after fewer UCMS exposures than in males (Fig. 5*g*, representative traces). At threshold current intensities, UCMS only displayed a trend to impact AP heights (Fig. 5*h*, *p *=* *0.058). Importantly, under suprathreshold stimulation, females in the 4 and 8 week UCMS groups showed significantly lower AP heights than controls for AP1 (4 weeks of UCMS, *p *=* *0.0084; 8 weeks of UCMS, *p *=* *0.022), AP2 (4 and 8 weeks of UCMS, *p *<* *0.0001), and AP3 (4 and 8 weeks of UCMS, *p *<* *0.0001), without differences between the UCMS exposures (Fig. 5*i*).

Additional assessments of hyperexcitability and adaptation examined maximum spiking frequency at rheobase, the lowest intensity current at which neurons begin to fire APs, and across all stimulus intensities (Table 6, statistics and effects). Spiking frequency at rheobase increased after UCMS, indicative of hyperexcitability, although *post hoc* testing yielded only trend-level differences between control and either 4 weeks (*p *=* *0.088) or 8 weeks (*p *=* *0.084) of UCMS. This may be because of different patterns displayed by female and male neurons, although there was no UCMS by sex interaction. Unlike spiking at rheobase, the maximum AP frequency across all stimulus intensities was significantly reduced after 8 weeks of UCMS in male PV^+^ neurons, suggesting that the strongest adaptation may occur in males experiencing 8 weeks of UCMS. AP threshold only demonstrated a trend toward reduction following UCMS, independent of sex, but this was not significant (*p *=* *0.087). Spike half-width was altered by UCMS in males but not females, with *post hoc* analysis via Tukey’s test showing a significant increase after 8 weeks of UCMS in males (*p *=* *0.0048 vs control), a feature suggesting slower AP recovery may be a male-specific adaptation.

## Discussion

This study shows distinct timelines for chronic stress effects on prefrontal PV^+^ neurons between male and female mice that parallel sex-specific vulnerability to stress-induced anxiety-like behaviors. Prefrontal PV^+^ neurons from male mice appear to require a longer exposure to chronic stress to affect their activity, coinciding with pronounced changes in behaviors. In females, a shorter exposure to chronic stress is sufficient to alter both PV^+^ interneuron activity, as measured by ΔFosB expression and excitability, and anxiety-like behaviors. However, driving the activity of prefrontal PV^+^ neurons in males with chemogenetics did not consistently enhance the expression of anxiety-like behaviors in response to UCMS, as we would have predicted whether PV^+^ neuron hyperactivity generates anxiety. These findings support the idea that prefrontal PV^+^ neurons are sensitive to chronic stress in a sex-specific manner, but how stress-induced changes in PV^+^ neuronal physiology play a role in sex-specific regulation of anxious behaviors in response to stress deserves further investigation.

### UCMS effects on medial prefrontal PV neuron activity parallel changes in behaviors

Reduced mPFC activity during stress is associated with vulnerability to anxiety-like and depressive-like behaviors (Vialou et al., 2014; Sinha et al., 2016). The mechanisms responsible for these stress-dependent dynamic changes have yet to be fully elucidated. Because prefrontal GABAergic interneurons tightly regulate the activity of principal projection neurons and are highly sensitive to stress (Maguire, 2014), they represent an ideal candidate to drive stress effects on prefrontal circuit activity. Here, we report and characterize, for the first time, changes in electrophysiological properties of prefrontal PV^+^ neurons that could underlie altered PFC network dynamics observed in chronic stress-related neuropsychiatric disorders. We also identified important sex differences that could increase vulnerability to stress-induced anxiety in females. Hyperexcitability followed by adaptation of IL layer II/III PV^+^ neurons developed in female neurons after 4 weeks of UCMS and persisted after 8 weeks of UCMS, paralleled by increased expression of ΔFosB, a marker of chronic activity, in PV^+^ neurons throughout the mPFC. In males, similar adaptations in the firing frequency of PV^+^ neurons and ΔFosB expression in IL layer were observed after only 8 weeks of UCMS. These findings align with previous work in males showing increased synaptic inhibition in IL layers after chronic variable stress (McKlveen et al., 2016) and dendritic hypertrophy of prefrontal interneurons after 21 d of restraint stress (Gilabert-Juan et al., 2013). The mechanisms underlying changes in the excitability of PV neurons in the PFC following chronic stress remain to be elucidated; however, previous work showed an increased number of glutamatergic VGlut1^+^ terminals on PV-expressing cells after chronic stress that could drive their hyperexcitability (Shepard and Coutellier, 2018). Changes in the excitation of PV neurons have been shown to induce plasticity of PV networks in the hippocampus: enhanced excitation of PV cells resulted in a high-PV network configuration (i.e., an increased proportion of PV neurons with high immunofluorescent signal) associated with reduced synaptic plasticity, which was also observed after an adverse, stressful event like fear conditioning (Donato et al., 2013). Other studies have identified glucocorticoid receptor-dependent mechanisms after chronic stress, leading to enhanced activity of PV-expressing cells in the hippocampus and PFC (Hu et al., 2010; McKlveen et al., 2016). While our current work does not provide insight into the underlying mechanisms, our data expand the understanding of stress-induced increases in prefrontal inhibitory transmission and identify the heightened sensitivity of female PV^+^ neurons to stress as a potential mechanism contributing to higher risk for stress-induced anxiety.

Indeed, changes at the cellular level were paralleled by sex-specific changes in anxiety-related behaviors, as previously described (Shepard et al., 2016; Page et al., 2019). While aspects of anxiety-like behaviors were affected by UCMS in both males and females (e.g., in the OF test), females displayed a wider range of behavioral alterations, as demonstrated in the EPM, marble-burying, and splash tests. The heightened behavioral response of females to stress could be the result of sex-specific chronic stress-induced dysregulation of the hypothalamic–pituitary–adrenal (HPA) axis. It has been established that after acute stress, females display a more robust neuroendocrine response and impaired negative feedback. However, the study of sex differences in HPA axis response to chronic stress remains limited, and further investigation is required (Heck and Handa, 2019). It is also possible that the heightened response of females to UCMS in the EPM and splash test is determined, in part, by their lower baseline anxiety-like and depressive-like behaviors, as previously reported (Walf and Frye, 2007; Eltokhi et al., 2020; Knight et al., 2021), while the elevated baseline anxiety-like and depressive-like behaviors in those tests in males could lead to a ceiling effect requiring more severe or longer stress exposure to induce significant changes. Others reported that lower baseline time and number of entries in the open arms of the EPM predict a highly susceptible phenotype (Nasca et al., 2019), which is contrary to our findings. However, this study focused on groups of male mice and susceptibility to a male-specific stress paradigm (social defeat stress), and not on baseline sex differences, which deserve further investigation.

We also observed that the heightened behavioral response of females to 4 weeks of UCMS is paralleled by higher levels of PV neurons expressing FosB in both IL and PrL layers. This could suggest that changes in both mPFC subdivisions after chronic stress could contribute to the stress-induced increase in anxiety-like behaviors seen in females. This would need to be tested in the context of the estrus cycle, as several studies have reported not only changes in baseline and stress-induced anxiety-like behaviors, but also changes in PV^+^ neurons, based on the phase of the female estrus cycle (Sayin et al., 2014; Ramos-Ortolaza et al., 2017; Clemens et al., 2019)

### Medial prefrontal PV neuron activation under baseline and stressful conditions impacts behaviors in males

Based on our prediction, we anticipated that the activation of prefrontal PV^+^ neurons during chronic stress exposure in males would enhance the impact of 4 weeks of UCMS on multiple behavioral end points, producing anxiety-like and depressive-like behaviors that resemble a female phenotype. Our findings indicate that, while chronic activation of prefrontal PV^+^ neurons did not affect behaviors in the open field test, UCMS interacted with chronic activation of prefrontal PV^+^ neurons in EPM and marble burying, the two anxiety-like behavior assays in which males showed resilience to 4 weeks of UCMS, as well as in the splash test, a measure of depressive-like behavior. However, differences were mostly noted between nonstressed and UCMS-exposed mice that received chronic activation of PV^+^ neurons, with hM3DGq/UCMS mice displaying higher anxiety than hM3DGq/nonstressed mice. This is in line with other work in male mice showing that chemogenetic inhibition of GAD-, somatostatin-, and PV-expressing neurons in the mPFC of male mice induced antidepressant-like effects (Nawreen et al., 2020; Fogaça et al., 2021), whereas negative allosteric modulators of GABA_A_ receptors prevented stress-induced anhedonia (Troppoli et al., 2022). While the lack of differences from the nonstress/control virus group renders the interpretation of results difficult, our findings could indicate that chronically activating prefrontal PV^+^ neurons decrease anxiety in nonstressful situations but increase anxiety when exposed to stress. This idea would need to be further investigated. Other studies recently reported that increasing inhibitory neurotransmission in the PFC rescued baseline anxiety-like and depressive-like behaviors in a mouse model of neurodevelopmental disorders (Yang et al., 2021), and early postnatal chronic activation of PV^+^ cells similarly decreased baseline anxiety-like behaviors in males tested in the EPM test but not in the OF test (Banerjee et al., 2022), suggesting that PV^+^ neurons could regulate specific aspects of anxiety-like behaviors in males under nonstressful conditions. This is in line with other reports showing that acute chemogenetic inhibition of somatostatin interneurons in the mPFC increased anxiety-like behavior in the EPM test, but not the OFT (Soumier and Sibille, 2014). The test-specific effects observed here and by others could reflect the important role of prefrontal inhibition in regulating specific aspects of anxiety. Indeed, it is usually considered that the OFT assesses trait anxiety, while the EPM assesses state anxiety (Jakovcevski et al., 2008; de Kort et al., 2021). The idea that activity of prefrontal PV^+^ neurons could regulate state, but not trait, anxiety is supported by the fact that anxiety-like behaviors were observed after 4 weeks of UCMS in the open field in males, without observable changes in PV^+^ neuron physiology, while anxiety-like behaviors were seen in the EPM test after only 8 weeks of UCMS, a time when we noticed increased numbers of PV/FosB neurons and altered excitability. Further investigations are, however, needed to draw definitive conclusions.

Our experimental design contains a few limitations that could be driving some observed effects. We cannot exclude the possibility that chronic activation of PV^+^ neurons leads to compensatory effects. However, this is unlikely, as 14 d of twice-daily CNO injection in PV:Cre mice expressing the hM3DGq virus did not change membrane properties or single AP characteristics of PV^+^ neurons (Stedehouder et al., 2018). Alternatively, the joint effect of pairing daily CNO injections with the mild stressor could enhance stressor severity enough to increase anxiety levels. Additionally, the half-life of CNO is shorter than those of some of the prolonged stressors used in our UCMS paradigm (Guettier et al., 2009; Purohit et al., 2018), and, therefore, the heightened activity of PV^+^ neurons did not fully overlap with those stress exposures. Finally, subdivisions of the mPFC, including the PrL and IL, have been shown to differentially regulate responses to stressful stimuli (Senn et al., 2014). Our DREADD injection targeted both mPFC subregions, in accordance with other reports showing that chronic activation of PV^+^ neurons in these regions increases anxiety-like behaviors in nonstressed females (Page et al., 2019). However, it might be the case that in males, exclusively activating IL, and not PrL, PV^+^ neurons, is necessary to heighten stress-induced anxiety, as suggested by the PV/FosB data indicating that only IL PV neurons are impacted by 8 weeks of UCMS. Finally, we cannot exclude the possibility that PV-dependent inhibition in the PFC regulates stress-induced increases in anxiety-like and depressive-like behaviors in a sex-specific manner. It was previously shown that positive modulation of α5 subunit-containing GABA_A_ receptors reduced stress-induced emotionality in female but not male mice (Piantadosi et al., 2016). This suggests that GABA dysregulation could contribute more to stress-related neuropathologies in females than in males.

### Possible mechanism of PV neuron firing adaptation

The observed changes in electrophysiological properties of prefrontal PV^+^ neurons after UCMS are consistent with intrinsic changes in the expression of ion channels involved in the establishment or postspiking re-establishment of the resting membrane potential. In response to prolonged depolarizing current steps, different classes of neurons display specific firing patterns, largely based on transmembrane sodium and potassium currents, and pathologic or physiological modulation of these ion channels can greatly change the firing behavior of a neuron (Bean, 2007). PV^+^ neurons generally display brief spikes; large, fast afterhyperpolarization; and sustained high-frequency firing with little or no adaptation across a wide range of current intensities (Tremblay et al., 2016). This classic phenotype was observed here for IL PV^+^ neurons from control male and female mice. The inability of IL PV^+^ neurons to maintain high-frequency firing after UCMS suggests adaptations in either sodium or potassium channel contributions to action potential firing and resetting, respectively. In some states of elevated neural activity *in vivo* or experimental administration of strong currents, some neurons eventually stop firing because of depolarization block, a state of silence at membrane potentials more depolarized (approximately –40 mV) than those that support the generation of action potentials (Bianchi et al., 2012). Depolarization block reflects reduced availability or persistent inactivation of voltage-gated sodium channels (Vilin and Ruben, 2001). Indeed, slow-opening and longer-lasting sodium channel inactivation emerges with sustained membrane depolarization, generating cumulative action potential adaptation and contributing to depolarization block (Qian et al., 2014). The possibility exists that the adaptation of PV^+^ neurons observed after UCMS is because of molecular alterations creating a permissive state in which depolarization block is more easily achieved, likely through changes in sodium channels. Such changes may also produce an elevation in the resting membrane potential, yielding easier conditions for achieving depolarization threshold and possibly earlier emergence of depolarization block. However, elevated resting membrane potentials were not consistent across all UCMS conditions that generated adaptation of AP firing, suggesting alternate molecular mechanisms likely underlie UCMS alterations in PV^+^ firing.

Another source by which UCMS may change AP firing is the modulation of potassium channel function. The Kv3 family of voltage-gated potassium channels is necessary to enable the fast-spiking phenotype of PV^+^ neurons (Kaczmarek and Zhang, 2017), and the increase in AP half-width in males supports altered Kv3 function. The adaptation in spiking observed here mimics the effect of genetic manipulations reducing Kv3.1 expression in dentate gyrus PV^+^ neurons or deleting Kv3.2 from deep-layer cortical interneurons (Lau et al., 2000; Medrihan et al., 2020). Reduced Kv3.1 expression in male hippocampus was reported after 21 d of chronic mild stress, without changes in frontal cortex expression (Chen et al., 2015). Kv3.1 has been associated with behavioral regulation in male mice: global Kv3.1 knockout reduced anxiety-like behaviors (Chow et al., 1999), while reduced Kv3.1 in dentate gyrus was associated with vulnerability to depression-like behaviors (Medrihan et al., 2020). However, the impact of UCMS on mPFC Kv3 channels and their involvement in modulating anxiety-like and depressive-like behaviors in both sexes remain unknown. Additionally, no change in AP half-width was observed after UCMS in female neurons, indicating that other molecular mechanisms governing AP generation in addition to Kv3 channels may be altered following UCMS, or that adaptations may differ by sex despite similar neural and behavioral outcomes. To date, sex differences in neuroadaptations remain understudied, and whether the divergent responses to UCMS by sex occur because of slower production of the same neuroadaptations in males and females or differential molecular changes by sex producing similar neural activity and behavioral phenotypes remains to be determined. Nonetheless, the current findings support a critical role for disrupted mPFC PV^+^ neuron-dependent circuit function in vulnerability to stress-induced anxiety, with voltage-gated sodium and Kv3 channels as possible sites of molecular adaptation. Since Kv3.1b, the main Kv3 family member expressed in layer II/III PV^+^ interneurons (Chow et al., 1999), is implicated in facilitating the fast-spiking phenotype of these neurons (Du et al., 1996), further exploration of the role of Kv3.1b in UCMS-induced adaptation and its regulation of behavior is warranted. It is interesting to note that, under basal conditions, Kv3.1 modulators have limited efficacy to disrupt AP firing, while they can restore pharmacologically or molecularly perturbed PV^+^ neuronal activity (Rosato-Siri et al., 2015; Andrade-Talavera et al., 2020). Thus, off-target effects of such treatments may be limited, further supporting Kv3.1-directed drugs as potential treatment targets to manage anxiety symptoms arising from chronic stress exposure.

Together, our results add to the accumulating evidence that enhanced inhibition in prefrontal circuits contributes to stress-induced anxiety-like and depressive-like behaviors (Gilabert-Juan et al., 2013; McKlveen et al., 2016; Nawreen et al., 2020; Fogaça et al., 2021). The present findings further demonstrate that prefrontal inhibitory transmission is more sensitive to stress in females than in males. The sex-specific temporal emergence of prefrontal PV^+^ neuroadaptations reported here likely contributes to the increased vulnerability of females to stress-induced anxiety.

## Synthesis

Reviewing Editor: Christie Fowler, University of California, Irvine Decisions are customarily a result of the Reviewing Editor and the peer reviewers coming together and discussing their recommendations until a consensus is reached. When revisions are invited, a fact-based synthesis statement explaining their decision and outlining what is needed to prepare a revision will be listed below. The following reviewer(s) agreed to reveal their identity: Gyorgy Lur, Mary (Molly) Heyer.

Both reviewers felt that the manuscript revisions significantly strengthened the conclusions. It was noted that the revisions were diligent and thorough, with rigorous statistical analyses. Thus, the manuscript has been greatly improved by the additional experiments, especially the additional behavioral testing and separate cohort of mice for immunofluorescence staining. The detailed discussion and interpretation of the data are now very interesting and balanced.

The novelty of the data was indicated as providing a detailed investigation into sex-specific differences in anxiety-like behaviors and mPFC PV+ neuron activity following chronic unpredictable mild stress in mice. This builds on prior work in this field and paves the way for future studies that may provide insight into sex-dependent therapeutic options for humans suffering from anxiety and depression.

However, several minor concerns remain that will need to be addressed in a revision:

1. The UCMS procedure should be written up in the Methods sections such that other groups will be able to follow it exactly in order to reproduce the findings. Please state precisely when and in what order the mice were exposed to each stressor. Either text or table format would be appropriate.

2. Please state the lux value of the lighting for the marble burying test, as this assay is particularly sensitive to light intensity.

3. Results line 293: do you mean mPFC PV expression or cFos expression in PV+ neurons as referred to in the Introduction?

4. Behavioral results should include a very brief interpretation, e.g. state how the results of a specific test are suggestive of an altered or unaltered anxiety-related behavior.

5. Please double check grammar throughout the manuscript e.g. Abstract line 11: four weeks of UCMS induces behavioral dysregulation in female mice and FosB activation in female mPFC PV neurons (the wording is that emotional dysregulation is in the female neurons) and Results line 413 should read: “Changes in PV+ interneuron excitability...align with sex differences”.
